# Changes in left ventricular blood flow during diastole due to differences in chamber size in healthy dogs

**DOI:** 10.1038/s41598-019-57180-6

**Published:** 2020-01-24

**Authors:** Katsuhiro Matsuura, Kotomi Sato, Kazumi Shimada, Seijirow Goya, Akiko Uemura, Takeshi Iso, Kana Yazaki, Zeki Yilmaz, Ken Takahashi, Ryou Tanaka

**Affiliations:** 1 Tokyo University of Agriculture and Technology, Department of Veterinary Surgery, 3-5-8 Saiwai-cho, Fuchu-shi, Tokyo 183-0052 Japan; 2 Teikyo University of Science, Department of Animal Science, 2-2-1, Senjyusakuragi, Adachi-ku, Tokyo 120-0045 Japan; 3 Juntendo University Graduate School of Medicine, Department of Pediatrics and Adolescent Medicine, 2-1-1 Hongo, Bunkyo-ku, Tokyo 113-8421 Japan; 4 Uludag University, Department of Internal Medicine, Özlüce Mahallesi, Veterinerlik Fak. Hayvan Hst., 16120 Nilüfer, Bursa Turkey; 5 Department of Veterinary Surgery, Tokyo University of Agriculture and Technology, Animal Medical Center, 3-5-8 Saiwaicho, 183-8509 Fuchu-shi, Tokyo Japan

**Keywords:** Heart development, Heart failure

## Abstract

Vorticity is a novel index that reflects diastolic function of left ventricle. The size of the ventricle can influence the ventricular diastolic blood flow. We evaluated effect of ventricular size on diastolic function and diastolic intracardiac blood flow using a particular species of dogs, which has a wide range of body size. Vector flow mapping was used for evaluation of intracardiac blood flow, and intraventricular pressure gradient (IVPG) was used for evaluation of diastolic function. 58 dogs weighing 1.3–42.3 kg were included in this study. Vorticity was found to be inversely proportional to the length of the ventricular chamber. Intraventricular pressure difference was positively correlated with the length of the left ventricle, whereas IVPG was not. This study showed that the vorticity is influenced by the size of the left ventricle independently of other factors. To evaluate the hemodynamic state of each individual appropriately by using vorticity and IVPD, ventricular size should be taken into account especially in the field of veterinary medicine and human pediatric and adolescent cardiology.

## Introduction

Diastolic heart failure is recognized as one of the major causes of congestive heart failure^1^. Pulsed-wave Doppler derived transmitral flow velocity patterns are commonly used for assessment of diastolic function. However, left ventricular diastolic dysfunction can display elevation of left atrial pressure, which can easily affect left ventricular inflow patterns^2^. Noninvasive method that is less load-dependent to assess diastolic function is strictly needed.

Vorticity can be obtained by intracardiac flow analysis using Vector Flow Mapping (VFM) or 4D-flow MRI^3,4^. Left ventricular vorticity during early diastolic phase reflect tau, which is the gold standard for measuring left ventricular relaxation property, independent from preload^5^. Another report showed that the vorticity related to clinical signs of diastolic dysfunction^5^. These reports suggested vorticity have a potential of to be a novel marker of diastolic function. “Also, VFM is promising method that it enables evaluation” of cardiac function from a new viewpoint particularly in evaluating the energy efficiency^6,7^. Intraventricular pressure gradient (IVPG) can be measured by analyzing color M mode Doppler images is used to evaluate suction force of left ventricle^8^, which closely correlated to vorticity^5^. As is said in IVPG in a previous report, intracardiac blood flow can be affected by chamber size of the ventricle^9^. In order to verify the utility of index derived from intracardiac flow analysis such as vorticity, it is necessary to elucidate the influence of the heart size on the intracardiac blood flow and clarify the causal relation between the heart size and the diastolic function. Understanding the relation between the heart size and intracardiac blood flow enables us to utilize vorticity especially in the field of veterinary medicine and human pediatric and adolescent cardiology.

It is difficult to elucidate the relationship between the heart size and the cardiac function by evaluating the cardiac function during the growth period over time in humans. Because the echocardiographic indices change as human grows. The changes in cardiac function during the growth period can be explained by the functional change due to the developing myocardial function rather than the change in the ventricular size^10^. Therefore, the study design using growing human is not suitable for the evaluating the contribution of chamber size to the cardiac function. Also, because cardiac anatomy and the conduction system can vary considerably between species^11^ and its effect on cardiac flow dynamics is unclear, comparative research using different species that have different heart sizes always make a question of how much the myocardial structure and conduction system differ from species to species. To remove specific differences, it is desirable to study a single species.

In the present study, we used dogs to examine the difference in diastolic blood flow depending on the heart size. Dogs are characterized by wide range of body size among the same species. For this reason, it is possible to compare the changes in intraventricular blood flow between a variety of heart sizes by comparing adult dogs of various body size. This study will show the effect of the heart size on the intraventricular blood flow and the diastolic function.

## Materials and Methods

### Animals

Client owned adult dogs referred to Tokyo University of Agriculture and Technology Animal Medical Center that had undergone echocardiographic examination and showed no obvious signs of cardiac disease were included. Dogs with hypertension and abnormal hydration status were excluded. The medical records of the dogs were reviewed, and clinical data including body weight, age, breed and conventional echocardiographic data were collected. Ethics Committee attached to Tokyo University of Agriculture and Technology Animal Medical Center approved this study. Owner’s consent was gained before each examination. The dogs were handled with the guidelines for the Institutional Animal Care and Use Committee of the Tokyo University of Agriculture and Technology.

### Echocardiographic examination

Echocardiographic examination was performed using a ProSound Alpha 10 ultrasonography system equipped with a 5 MHz sector probe (Hitachi-Aloka Medical, Co. Ltd., Tokyo, Japan). All examinations were performed on non-sedated awake dogs. Cardiac cycles were recorded for each plane at the end of the expiratory phase. Left ventricular inner diameter at diastole (LVIDd), left ventricular inner diameter at systole (LVIDs), ejection fraction (EF) and fractional shortening (FS) were obtained from left ventricular short axis M-mode images. Peak E wave velocity (E-vel), peak A wave velocity (A-vel) and E/A ratio were measured from the pulse-wave Doppler images of the mitral inflow. The peak myocardial velocity during early diastole (e′) and late diastole (a′) at lateral annulus and septal annulus were measured, respectively, using tissue Doppler imaging. The length of the left ventricle (LVL) was measured as the distance between the mitral annulus to LV apex. Sphericity index (SI) was calculated with LVIDd divided LVL. For acquisition of the data for VFM analysis, all dogs were connected to an ECG and placed in a recumbent position on the left side. Two-dimensional movies of the left parasternal long axis five chamber view were recorded. The color area was adjusted so as to include the inflow tract, the entire lumen and the outflow tract of the left ventricle. The Nyquist limit was maximized to minimize aliasing phenomenon. At least three images were recorded for each dog.

### Vector flow mapping

Obtained data were processed using software (DAS-RS1, Hitachi-Aloka Medical, Co. Ltd.,Tokyo, Japan) to acquire the velocity vector fields in the left ventricle. Briefly, manual tracing in the first frame of the endocardial border of LV followed by speckle tracking was applied to determine the cardiac wall motion of all the frames. The software computed the velocity vector fields from the color Doppler and LV wall motion information using a system established by Itatani *et al*.^4^. The aliasing was corrected by color baseline shift when the aliasing phenomenon was recognized. Based on VFM images, the vorticity and intracardiac energy loss (EL) of the region of interest were automatically calculated as previous study by Goya *et al*.^12^.

### Intraventricular pressure gradient (IVPG)

IVPD was calculated using in-house code written in MATLAB (The MathWorks, Natick, MA) as previously described^13^. Briefly, for the analysis of IVPG, color M-mode apical four chamber views were stored. The cursor of M-mode was set parallel to mitral inflow.

1 $$(\partial {\rm{P}})/(\partial {\rm{s}})=-\,\rho ((\partial v)/(\partial t)+{\rm{v}}(\partial v)/(\partial s)).$$

In Eq. (1), P is the pressure, ρ is the constant blood density, v is the velocity, s is the position along the color M-mode line, and t is the time. The Euler Eq. (1) was used to calculate the intraventricular pressure difference (IVPD) at each point. The temporal relative pressure of each part of interest was obtained by calculating the line integral along the scan line. This protocol was previously validated against direct measurements using micromanometers^8^. IVPG is used rather than IVPD when there is a great difference in the heart size of each individual. IVPG was calculated by dividing the IVPD by the LVL. To evaluate the segmental pressure difference of each part of the heart, the left ventricle was trisected as previously described^13^. The apical side of the heart was named “apical”, the middle part was named “mid”, and the basal part was named “basal”. IVPG data was obtained after conventional echocardiography in the same situation. The average of the calculated values of three heartbeats was taken as the measurement data.

### Statistical analysis

The statistical analyses were performed using R software version 3.3.2. The significance level was set at p < 0.05. The Vorticity and EL values were logarithmically transformed for the statistical analysis. Grubbs test was used for the outlier test. If outliers were present, they were removed. Pearson’s correlation coefficients were used to evaluate correlations between vorticity, EL, IVPD, IVPG and echocardiographic parameters. Multivariate linear regression analysis was performed to determine the independent variables that correlate with vorticity and EL. A stepwise method was used for selection of the variables, choosing the variables which minimized Akaike’s information criterion.

## Results

### Study population

The number of cases was 58. The mean weight was 8.65 (1.3–42.3, SD 6.93) kg. The length of the left ventricle was 34.0 (21.9–49.0, SD 7.3) mm on average. The mean heart rate was 117.6 (66.0–187.5, SD 24.5) bpm. In this study, 10 Mongrel dogs, 10 Miniature Dachshunds, 9 Toy Poodles, 7 Beagles, 3 Chihuahuas, 3 Labrador Retrievers, 2 Pomeranians, 2 American Cocker Spaniels and one from each of the following breeds: Border Collie, Boston Terrier, Chinese Crested dog, Cavalier King Charles Spaniel, English Cocker Spaniel, French Bulldog, Miniature Schnauzer, Maltase, Papillon, Shiba Inu and Yorkshire terrier were included.

The echocardiogram data were shown in the Table 1. Table 2 showed data obtained by vector flow mapping analysis and intraventricular pressure difference analysis.

**Table 1:** Conventional echocardiographic parameters.

Parameters		mean		SD
LVIDd	(mm)	25.9	±	7.4
LVIDs	(mm)	14.3	±	5.7
EF	(%)	82.6	±	9.1
FS	(%)	46.1	±	10.7
E vel	(cm/sec)	66.4	±	16.1
A vel	(cm/sec)	67.6	±	16.5
IRT	(sec)	51.8	±	20.1
e′	(cm/sec)	7.82	±	2.00
E/e′		8.96	±	2.27
E/A		0.98	±	0.26

LVIDd: left ventricular inner diameter at end-diastole, LVIDs: left ventricular inner diameter at end-systole, EF: ejection fraction, FS: fractional shortening, E-DcT: E wave deceleration time, IRT: isovolumic relaxation time.

**Table 2:** Parameters obtained from VFM, IVPD and IVPG analysis.

Parameters		mean		SD
Vorticity	(1/s)	396.4	±	134.0
Energy Loss	(J/(m • s))	38.8	±	23.5
Total IVPD	(mmHg)	2.86	±	0.92
Basal IVPD	(mmHg)	1.13	±	0.39
Mid IVPD	(mmHg)	1.41	±	0.47
Apical IVPD	(mmHg)	0.33	±	0.25
Total IVPG	(mmHg)	0.94	±	0.30
Basal IVPG	(mmHg)	0.36	±	0.12
Mid IVPG	(mmHg)	0.44	±	0.14
Apical IVPG	(mmHg)	0.11	±	0.09

VFM: vector flow mapping, IVPD: intraventricular pressure difference, IVPG: intraventricular pressure gradient.

### Characteristics of the canine heart with various size

**Figure 1:**
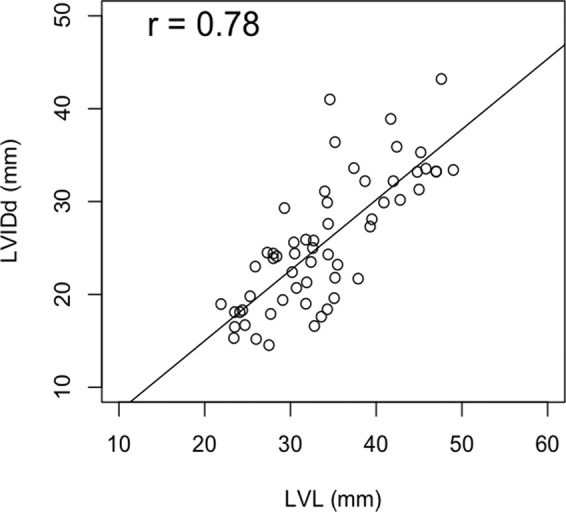
The relationship between short and long axis diameter. LVL was linearly correlated with LVIDd (R = 0.78, p < 0.01).

The mean value of the short axis inner diameter of the left ventricle (LVIDd) was 25.90 (14.5–43.2, SD 7.36) mm. The sphericity index (SI) of the left ventricle was 0.75 (0.51–1.0, SD 0.12). The relationship between short and long axis diameter was shown in Fig. 1. LVL was linearly correlated with LVIDd (R = 0.78, p < 0.01). Statistical correlation was not found between LVL and SI.

Conventional indexes of diastolic property (E vel, e′, E/A, E/e′) were not statistically correlated with LVL.

### Relationship between the size of the left ventricle and the vorticity

**Figure 2:**
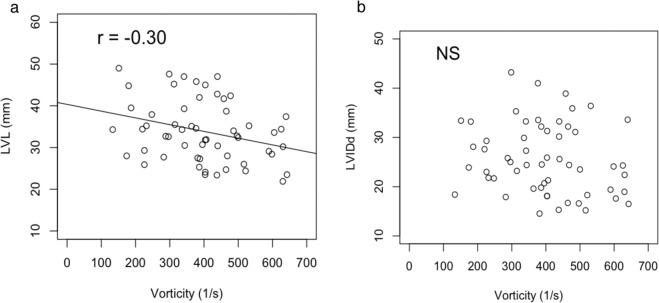
(**a**) Correlation between vorticity and LVL: longitudinal length of left ventricle, from endocardial border of the apex to midpoint of the mitral annulus. Significant negative correlation between LVL and vorticity (R = −0.30, p < 0.05) was observed. (**b**) Correlation between Vorticity and LVIDd: shot axis left ventricular inner diameter during diastole. Statistical correlation was not seen between vorticity and LVIDd.

**Figure 3:**
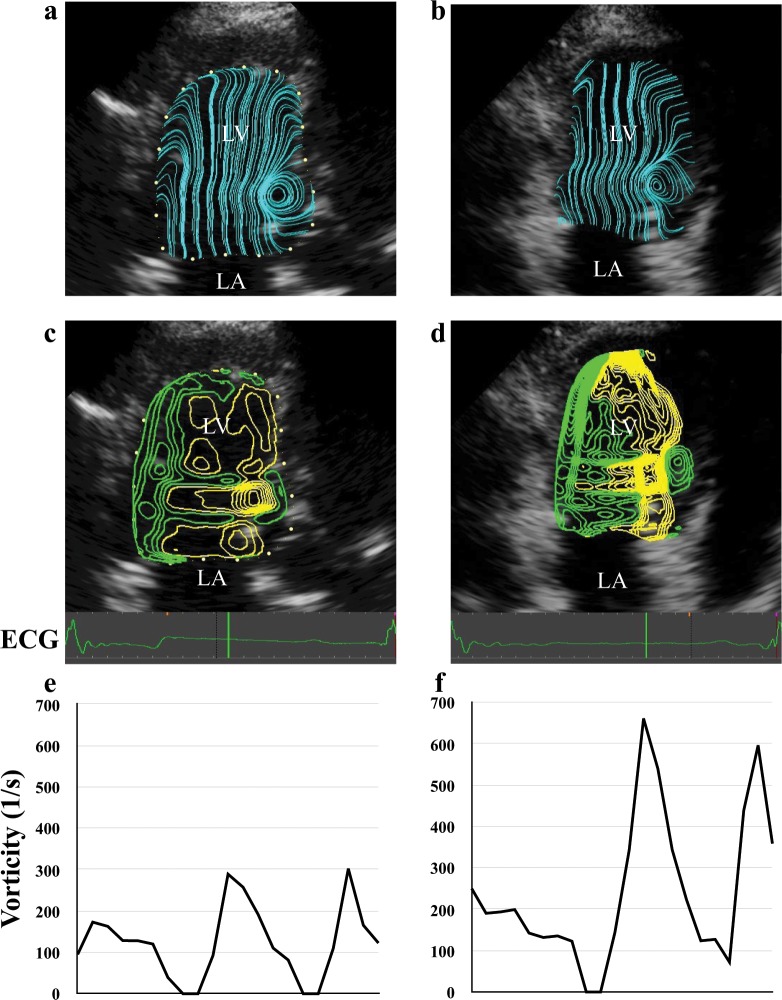
The analysis results of 2 healthy dogs. The left sided figures (**a**,**c**,**e**) are VFM derived data of medium-sized dog weighing 14 kg and 45 mm length of LV. The right sided figures (**b,d,f**) are data of a small dog weighing 3 kg and 22 mm of LV length. (**a,b**) visualize the stream line at early diastolic phase with the highest vorticity in the left ventricle. A vortex on the tip of the anterior mitral leaflet is visible in the left ventricle of both cases. (**c**,**d**) Show the contours of vorticity in the same time phase as the upper figures. Vorticity was highest at the location of the vortex seen in the figure above. The smaller heart on the right side has denser contours, representing higher vorticity. (**e**,**f**) Are the transition of the vorticity in a cardiac cycle. Diastolic vorticity is higher in the small heart on the right side. LV, left ventricle; LA, left atrium.

Significant negative correlation between LVL and vorticity (R = −0.30, p < 0.05) was observed, but correlation with LVIDd and SI were not (Fig. 2). A typical example of the difference in vorticity between a large heart and a small heart was shown in Fig. 3.

### Relationship between LVL and IVPD, IVPG

**Figure 4:**
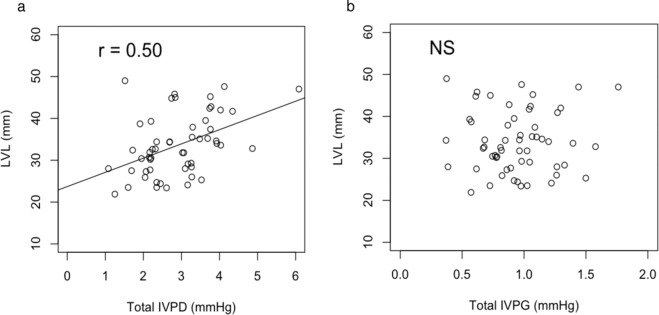
(**a**) Correlation between TIVPD: total intraventricular pressure difference and LVL: longitudinal length of left ventricle, from endocardial border of the apex to midpoint of the mitral annulus. Significant positive correlation between TIVPD and LVL (R = 0.50, p < 0.05) was observed. (**b**) Correlation between TIVPG: total intraventricular pressure gradient and LVL. Statistical correlation was not seen between TIVPG and LVL.

LVL showed a significant positive correlation with total IVPD (R = 0.50, p < 0.01) and IVPD of each part (BIVPD; R = 0.36, p < 0.01, MIVPD; R = 0.41, p < 0.01, AIVPD; R = 0.32, p < 0.05), whereas no correlation was found between LVL and IVPG (Fig. 4).

### Multivariable regression analysis

According to the multivariate analysis, the independent predictors of vorticity were LV length and E-vel. A significant regression equation was found (F (3, 46) = 12.75, p < 0.001), with an adjusted R2 of 0.41. Participants’ predicted log_10_Vorticity was equal to −0.3074 × LV-length + 0.3345 × E-vel + 0.3795 × MIVPG.

VFM derived vorticity was identified as independent predictor of MIVPG. A significant regression equation was found (F (1, 48) = 19.91, p < 0.01), with an adjusted R^2^ of 0.2784. Participants’ predicted MIVPG was equal to 0.5414 × log_10_Vorticity.

Transmitral peak E wave velocity and MIVPG were identified as independent predictors of peak EL. A significant regression equation was found (F (2, 45) = 26.11, p < 0.01), with an adjusted R^2^ of 0.5165. Participants’ predicted log_10_peak EL was equal to 0.4801 × E-vel + 0.3934 × MIVPG.

## Discussion

We evaluated the effect of ventricular size on diastolic function and diastolic intracardiac blood flow using dogs, which had a diversity of body size. Sparse relation between SI and LVL implies that size of the heart did not dramatically affect shape (fatness or thinness) of the heart in dogs.

As with cardiac function, conventional diastolic indexes were not correlated with the chamber size of the heart. These characteristics of the canine heart allowed us to simply consider the impact of the size of the ventricular chamber on intracardiac flow.

Vortex is reported to be smaller in a pathologically dilated heart^14^. However, it is not clear how the size of the heart affects vortex formation and dynamics, because the function of the heart is different between various sized diseased heart. In the present study, we showed that the longitudinal size of the healthy left ventricle was inversely correlated with the strength of vortex in the left ventricle independent from other factors. A correction of vorticity approximately 50 (1/s) per 1 mm LV length may be necessary in the 2–5 cm LVL range. According to the study using the volume overloading model by Pasipoularides^15^, strength of the vortex in the right ventricle was attenuated as dilatation of the ventricle progressed. He explained the reason by using the concept of convective deceleration load (CDL). When blood flows into the larger ventricle, CDL is greater than in the small heart and mitral inflow is attenuated by CDL. In this study, IVPG equivalent to CDL was relatively insensitive to changes in heart size. This indicates that in a healthy heart, CDL cannot explain the relationship between ventricular size and blood flow. The reason why the longitudinal size of the heart affects the vortex remains unclear. Hydrodynamic computational simulation may be useful to further verify this phenomenon.

In the present study, the independent predictors of vorticity other than LV length was peak E wave velocity. Both the vortex and transmitral flow are considered to be influenced by ventricular relaxation^5^, and it is not surprising that the strength of the vortex correlates with the peak E wave velocity in the present study as also reported in study in humans^16^. On the other hand, the relationship between vorticity and peak E can change depending on the pathological conditions. When a relaxation abnormality is observed, the vortex is strongly correlated with the E wave^16^. However, in the volume overloading model dogs, despite the inflow velocity was higher, the obtained vortex strength was weaker than in the normal dog^14^. They explained that the cardiac dilatation due to the volume overload made stronger impact to the strength of the vortex than the ventricular inflow velocity. To further verify the relationship between vorticity and mitral inflow velocity, an additional clinical study including pathological state is warranted.

### IVPG and IVPD

IVPG is an important factor of left ventricular relaxation ability and is reported to be associated with diastolic heart failure^17^. As we showed, within a single species of animal, IVPD was positively correlated with LV length and IVPG was not. Because IVPD is susceptible to the heart size even within the single species, IVPG should be used when comparing the groups of various heart sizes in the field of veterinary medicine and human pediatric/adolescent cardiology. Popovic considered that small animals such as rats and mice might require a larger IVPG to keep the filling pressure within short RR intervals^9^. In our study, the change of IVPG by the size of the heart was not seen using a single species. Additionally, HR was not selected as a predictor of IVPG in the multivariate analysis. It was presumed that the IVPG is larger in smaller animals because it reflects the difference in baseline cardiac function due to the difference in species in the previous study.

### Energy loss

From a new viewpoint of the energy efficiency, EL was not correlated with the size of the left ventricle, unlike vorticity. The larger the E wave and the IVPG, the greater the energy loss in the left ventricle. This is consistent with a previous study that suggested EL is affected by the E wave, which is an indicator of preload^18^. On the other hand, in this study, EL was correlated with IVPG, which is an index of relaxation. However, because the target group in this study did not include individuals with pathological relaxation ability, further research is needed to clarify its clinical significance.

## Conclusion

We showed that the strength of the vortex is influenced by the size of the left ventricle independently of other factors. We should take this consideration into account to evaluate the hemodynamic state of each individual appropriately by using vorticity and IVPD especially in the field of veterinary medicine and human pediatric and adolescence cardiology.
